# Contingency in the convergent evolution of a regulatory network: Dosage compensation in *Drosophila*

**DOI:** 10.1371/journal.pbio.3000094

**Published:** 2019-02-11

**Authors:** Christopher Ellison, Doris Bachtrog

**Affiliations:** Department of Integrative Biology, University of California Berkeley, Berkeley, California, United States of America; Ludwig Maximilian University of Munich, Germany

## Abstract

The repeatability or predictability of evolution is a central question in evolutionary biology and most often addressed in experimental evolution studies. Here, we infer how genetically heterogeneous natural systems acquire the same molecular changes to address how genomic background affects adaptation in natural populations. In particular, we take advantage of independently formed neo-sex chromosomes in *Drosophila* species that have evolved dosage compensation by co-opting the dosage-compensation male-specific lethal (MSL) complex to study the mutational paths that have led to the acquisition of hundreds of novel binding sites for the MSL complex in different species. This complex recognizes a conserved 21-bp GA-rich sequence motif that is enriched on the X chromosome, and newly formed X chromosomes recruit the MSL complex by de novo acquisition of this binding motif. We identify recently formed sex chromosomes in the *D*. *melanica* and *D*. *robusta* species groups by genome sequencing and generate genomic occupancy maps of the MSL complex to infer the location of novel binding sites. We find that diverse mutational paths were utilized in each species to evolve hundreds of de novo binding motifs along the neo-X, including expansions of microsatellites and transposable element (TE) insertions. However, the propensity to utilize a particular mutational path differs between independently formed X chromosomes and appears to be contingent on genomic properties of that species, such as simple repeat or TE density. This establishes the “genomic environment” as an important determinant in predicting the outcome of evolutionary adaptations.

## Abstract 2

A study of Drosophila species with independently formed sex chromosomes reveals that diverse mutational paths are utilized to evolve hundreds of de novo binding motifs for the dosage compensation complex; the “genomic environment” is an important determinant in the outcome of evolutionary adaptations.

## Author summary

We address how predictable evolution is at the DNA sequence level in nature by studying the parallel evolution of a phenotype that is well understood at the molecular level: the acquisition of sex chromosome dosage compensation in fruit flies. While female flies have two X chromosomes, the males have to compensate by up-regulating genes on their single X chromosome. They do this by using specific sequence motifs on the X chromosome to recruit a cluster of proteins and RNAs called the male-specific lethal (MSL) complex. However, different fruit fly lineages have independently co-opted the MSL complex to dosage compensate newly formed X chromosomes through the acquisition of hundreds of new binding sites, thereby providing independent replicas of the evolutionary process both within and between species. Moreover, dosage compensation has often evolved very recently, allowing us to infer the causative mutations by which the novel binding motifs arose. Genome sequencing and genomic occupancy maps of the MSL complex allowed us to infer the location of novel binding sites on the recently formed sex chromosomes of flies from the *Drosophila melanica* and *D*. *robusta* species groups. We show that species use diverse mechanisms to generate novel MSL-binding sites, including the use of presites, expansion of runs of simple sequences, or insertion of transposable elements. We also show that the propensity for using different types of mutations differs between lineages and depends on genomic properties of a species.

## Introduction

What would happen if we “replay the tape of life” [1]? The question of whether adaptation follows a deterministic route largely prescribed by the environment or whether evolution is fundamentally unpredictable and can proceed along a large number of alternative trajectories has until recently been a fascinating problem that could not be addressed directly.

In the past decade, however, advances in DNA sequencing technology have allowed researchers to tackle this question using two complementary approaches. Experimental evolution of viruses, bacteria, and yeast, in combination with genome sequencing, has allowed direct identification of adaptive mutations in order to address the relative contributions of determinism and stochasticity in the evolutionary process [2]. Genomic analysis of populations experimentally evolved under controlled laboratory conditions has consistently revealed parallelism in which mutations in certain genes are repeatedly selected [3,4]. These studies are typically limited to systems that can be rapidly propagated in the lab and many relevant evolutionary parameters (such as environment, population size, etc.) are controlled by the experiment, and their applicability to natural systems is sometimes unclear [5].

Studies of parallel adaptations in the wild are a complementary approach to understanding the repeatability of evolution [2]. Organisms evolving under similar ecological conditions often evolve similar traits, and striking examples of genetic convergence at the DNA level have been recently discovered. For example, plant-feeding insects have independently and repeatedly colonized many different plant taxa, and highly diverged insect orders have evolved cardenolide resistance through the exact same amino-acid substitution in Na^+^/K^+^-ATPases [6]. Similarly, three distantly related lineages of snakes have convergently evolved resistance to the tetrodotoxin found in their prey via the same amino-acid mutation in a voltage-gated sodium channel [7]. Phenotypic convergence can also result from noncoding changes. Parallel evolution of trichome patterning in *Drosophila* [8] or wing pattern mimicry in *Heliconius* butterflies [9] both involved regulatory mutations that altered the expression pattern of a transcription factor. The parameters of convergent evolution in protein-coding genes are fairly well understood and often involve a small number of amino-acid mutations of large effect size that are constrained to specific regions of the protein because of pleiotropy. By contrast, how changes in *cis*-regulatory regions contribute to convergence is less well understood and hampered by our limited understanding of the global *cis*-regulatory structure of a phenotype [10]. Convergent regulatory evolution involves a much larger set of mutational targets and mechanisms: A single regulatory mutation affecting a transcription factor could act in *trans* to change the expression pattern of a suite of target genes (as observed in *Drosophila* and *Heliconius*) or multiple independent *cis*-acting mutations could act in concert to produce the selected phenotype. Furthermore, these regulatory mutations can arise via a large variety of mechanisms, from transposable element (TE) insertions to microsatellite expansions, and identifying causative adaptive mutations in nature is challenging, especially at noncoding DNA [10]. Moreover, adaptation to novel habitats often involves multiple selective agents whose relative importance is often unclear, making interpretations of convergent evolution (or a lack thereof) challenging [2].

**Fig 1:**
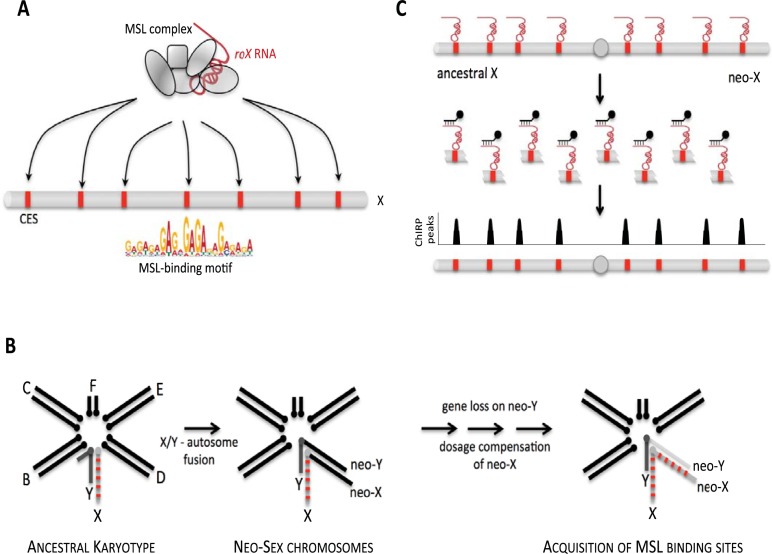
Dosage compensation and neo-sex chromosomes in *Drosophila*. (A) MSL-mediated dosage compensation in *Drosophila*. The MSL complex consists of several proteins and noncoding RNAs (*roX* RNAs) and targets the X chromosome at CESs that contain the MSL-binding motif (as GA-rich sequence motif). (B) Formation of neo-sex chromosomes in *Drosophila*. The ancestral karyotype of *Drosophila* consists of five large rods (the ancestral X, which is conserved across *Drosophila*, and the autosomal arms Muller element B, C, D, and E) and the small dot chromosome (Muller element F). Autosomes repeatedly fused to the sex chromosomes, forming neo-X and neo-Y chromosomes. Loss of genes on the neo-Y creates selective pressure to dosage compensate neo-X genes and has repeatedly evolved in *Drosophila* by co-opting the MSL complex through the acquisition of novel MSL-binding sites. (C) ChIRP can be used to identify MSL-binding sites on *Drosophila* sex chromosomes. The *roX* RNA is bound in vivo to CESs; chromatin is cross-linked and fragmented, and *roX2* is affinity purified and sequenced. CES, chromatin entry site; ChIRP, Chromatin Isolation by RNA Purification; MSL, male-specific lethal.

Here, we address how predictable evolution is at the DNA sequence level in nature by studying the parallel evolution of a regulatory phenotype that is well understood at the molecular level: the acquisition of dosage compensation in fruit flies. Many species with heteromorphic sex chromosomes have evolved mechanisms to equalize the amount of gene product from the X chromosome in males and females [11]. In *Drosophila*, males compensate for reduced dosage of X-linked genes by hypertranscribing their hemizygous X chromosome through epigenetic modifications [12]. At the molecular level, this is achieved by recruiting the male-specific lethal (MSL) complex to numerous chromatin entry sites (CESs) on the X in a sequence-specific manner [13] (**Fig 1A**). The MSL complex targets a 21-bp long, GA-rich sequence motif that is enriched on the X chromosome (the MSL recognition element, or MRE) [13]. The complex then spreads from CESs to the rest of the X and induces chromosome-wide hyperacetylation of H4K16, which results in up-regulated transcription on the X chromosome [14].

MSL-mediated dosage compensation evolved over 60 million years (MY) ago and is conserved across *Drosophila* species [15,16]. However, different species in this genus co-opted the MSL machinery to evolve dosage compensation on newly evolved neo-sex chromosomes [15,16]. In particular, fusions between the ancestral sex chromosomes (that is, the original X and Y chromosome shared by all members of the genus *Drosophila*) and autosomes have repeatedly created so-called neo-sex chromosomes [17]. Once fused, the neo-sex chromosomes follow a distinct evolutionary trajectory over tens of millions of years until they obtain the classical properties of ancestral sex chromosomes: The neo-Y chromosome degenerates as its protein-coding genes are inactivated, and the neo-X chromosome is up-regulated to compensate for this gene dosage imbalance [12,18]. During this transition, the age of the neo-sex chromosomes broadly correlates with their level of differentiation.

Dosage compensation evolves on newly formed X chromosomes by co-opting the MSL complex through the acquisition of new MSL-binding sites (**Fig 1B**). We recently studied the evolution of MSL-binding sites in *D*. *miranda*, a model species for sex chromosome evolution that possesses two neo-X chromosomes that were formed about 13–15 MY and 1.5 MY ago, respectively [19,20]. We found that diverse mutational paths contributed to MSL-binding–site evolution [21], but the majority of novel MSL sites on the younger neo-X were created by insertions of a domesticated helitron TE containing the GA-rich sequence motif recognized by the MSL complex (the MRE) [22,23]. We also detected highly eroded remnants of a related TE at the much older neo-X chromosome of this species, in which dosage compensation evolved around 13–15 MY ago [22].

Independently formed neo-X chromosomes are faced with the same evolutionary challenge: to co-opt the existing MSL machinery and up-regulate hundreds of genes simultaneously in response to neo-Y degeneration. This creates a set of fascinating questions: How are new binding sites acquired on different positions along the neo-X chromosome of a lineage or on independently evolved neo-X chromosomes between species? Does evolution predominantly follow the same molecular path to evolve new MSL-binding sites, do species-specific solutions evolve to the same problem, or do independent binding sites evolve by diverse molecular mechanisms even within a lineage?

To address these questions, we use comparative genomic and functional analysis to infer the mutational path evolution has taken to acquire novel MSL-binding sites. We focus on *Drosophila* species in the *D*. *melanica* and *D*. *robusta* groups, which are promising new systems to study the independent rewiring of the MSL complex. Cytogenetic studies have shown that species in this clade have independently evolved neo-sex chromosomes [24], thus ensuring that dosage compensation evolved in parallel for these species. Phylogenetic dating suggests that neo-sex chromosomes in this group are young [24], which should allow us to identify the causative mutations that created novel MSL-binding sites on neo-X chromosomes.

In this study, we generate genomic data for five species from the *D*. *melanica*/*D*. *robusta* groups to identify the specific X-autosome fusions in these species and date their formation. We create maps of MSL occupancy for three species in which dosage compensation on the neo-X has evolved independently and recently, using Chromatin Isolation by RNA Purification (ChIRP; see **Fig 1C**). Comparative analysis allows us to reconstruct the mutations generating novel MSL-binding sites, and we infer both heterogeneity and convergence of binding site evolution within and between species. Our results demonstrate that evolution is highly opportunistic yet contingent on the genomic background. We show that species use a diverse spectrum of mutational events to generate novel MSL-binding sites, but the propensity for different types depends on genomic contingencies of a species.

## Results

### Identification of independently formed neo-sex chromosomes in *Drosophila*

**Fig 2:**
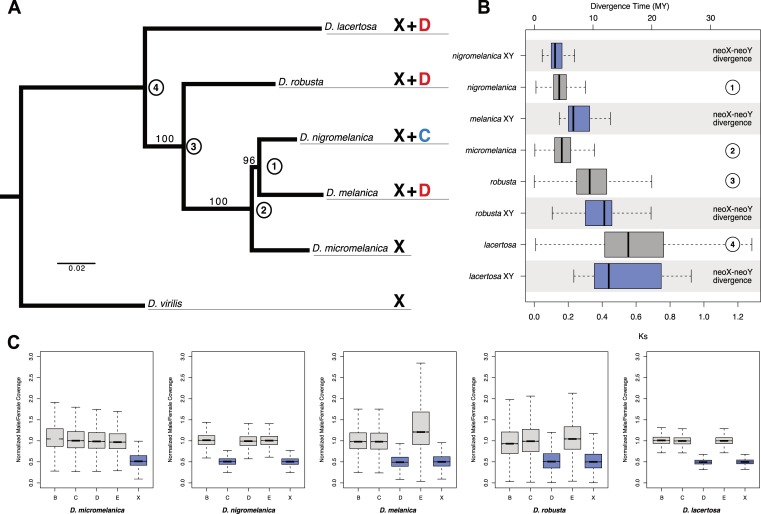
Phylogenetic relationships and karyotype evolution in the *D*. *melanica* and *D*. *robusta* species groups. (A) Whole-genome maximum likelihood phylogeny inferred using the RAxML rapid bootstrapping algorithm. Bootstrap support values are shown on branches, and nodes are numbered for reference to Panel B. Each species is labeled according to which chromosomes are X linked. X refers to the ancestral X chromosome, while the other chromosomes are referred to as Muller elements B–E (the small dot chromosome, Muller F, is not shown). (B) Divergence (calculated as Ks and converted to MY ago) between neo-X/Y chromosomes as well as the nodes labeled in Panel A. Boxes reflect the distribution of Ks values for all neo-X/Y gene pairs or between-species orthologs. Underlying data can be found in S1 Data. (C) X-linkage was determined based on the ratio of male/female Illumina sequencing coverage for genomic libraries aligned to the female assemblies (autosomes = gray, X chromosomes = blue). Underlying data can be found in S2 Data. MY, million years.

Young neo-sex chromosomes of *Drosophila* have formed independently by fusions between autosomes and the ancestral sex chromosomes [12]. Over time, they acquire the stereotypical properties of the ancestral X and Y, and their repeated formation in different lineages at different time points allows us to contrast neo-sex chromosomes at various stages of differentiation [25,26]. Cytogenetic comparisons suggest that neo-sex chromosomes have evolved independently multiple times in species from the *virilis*–*repleta* radiation [24], but their neo-sex chromosomes were not examined at the genomic level. To identify neo-sex chromosomes and infer their evolutionary history, we performed Illumina whole-genome sequencing of males and females from five species in the *D*. *robusta*/*D*. *melanica* sister groups (**Fig 2; S1 Table**). We generated de novo assemblies from the female sequencing data and created a whole-genome alignment to identify orthologous regions between all five species, which we used to infer their phylogeny. Our genome-wide phylogenetic analysis confirms previous inferred relationships among members of these groups based on a handful of genes [24,27], with *D*. *melanica* and *D*. *nigromelanica* being sister species and *D*. *micromelanica* as their outgroup (forming the *melanica* group), whereas *D*. *robusta* and *D*. *lacertosa* are more distantly related (**Fig 2A**). We used sequence divergence between species pairs to roughly date their split times. Assuming a neutral mutation rate of 3.46 × 10^‐9^ per year [28], we estimate that species from the *D*. *melanica* subgroup split very recently; *D*. *melanica* and *D*. *nigromelanica* diverged roughly 4.3 MY ago, *D*. *robusta* split from the *D*. *melanica* species about 9.4 MY ago, and *D*. *lacertosa* diverged 16 MY ago (**Fig 2B**).

Neo-sex chromosomes were previously reported for four of the five species investigated here, with *D*. *micromelanica* lacking an X-autosome fusion. The neo-sex chromosomes of *D*. *nigromelanica* and *D*. *melanica* were thought to be homologous [24], while X-autosome fusions occurred independently in *D*. *robusta* and *D*. *lacertosa* [24]. We used male and female genomic coverage data to infer which autosomal chromosome arm became the neo-sex chromosome in each species and to estimate the date at which the fusion occurred (see Materials and methods). As expected, we identified the ancestral X chromosome (Muller element A, which is shared across *Drosophila*) by reduced male coverage in each species, and we confirmed the presence of a neo-X chromosome in *D*. *nigromelanica*, *D*. *melanica*, *D*. *robusta*, and *D*. *lacertosa*, as well as the absence of a neo-X in *D*. *micromelanica* (**Fig 2C**). Intriguingly, however, we find that a different chromosome arm formed the neo-sex chromosome in the *melanica* group: Muller element D (corresponding to chromosome 3L in *D*. *melanogaster*) became the neo-sex chromosome of *D*. *melanica*, while Muller element C (chromosome 2R in *D*. *melanogaster*) formed the neo-sex chromosome of *D*. *nigromelanica*. Thus, contrary to parsimonious interpretations based on cytological data, our genomic comparison shows that neo-sex chromosomes formed independently at least two times in the *melanica* group and imply that they are younger than previously assumed. We also confirmed that Muller element D (chromosome 3L) became the neo-sex chromosome of both *D*. *lacertosa* and *D*. *robusta* (**Fig 2C**), but the lack of X-autosome fusions in multiple species of the *lacertosa* and *robusta* subgroups indicates that these fusions originated independently [24]. Muller element D has become sex–linked multiple times in the *Drosophila* genus and in Diptera [29], suggesting that this chromosome may have an intrinsic propensity to become a sex chromosome.

### Dating of neo-sex chromosome formations

The age of each species group with unique or shared neo-sex chromosomes sets a limit to the age of their chromosomal fusions (**Fig 2B**). Phylogenetic analysis suggests that *D*. *melanica* and *D*. *nigromelanica* diverged about 4.3 MY ago, implying that both species groups’ neo-sex chromosomes are younger than that age. Sequence divergence of homologous neo-sex–linked genes provides an independent estimate of their age: older neo-Y chromosomes harbor fewer genes (and fewer neo-X/neo-Y gene pairs), and levels of sequence divergence between orthologous gene pairs increases with the age of the neo-sex chromosome. We identified 118 pairs of homologous neo-sex–linked genes in *D*. *nigromelanica*, 20 in *D*. *melanica*, 16 in *D*. *robusta*, and 11 in *D*. *lacertosa*, and sequence divergence between homologous gene pairs increases with decreasing gene number (**S2 Table, Fig 2B**). This analysis suggests that *D*. *nigromelanica* has a relatively young neo-sex chromosome (mean synonymous site divergence [dS] = 0.16, 4.6 MY), followed by *D*. *melanica* (mean dS = 0.26, 7.5 MY), while the sex chromosomes of *D*. *robusta* and *D*. *lacertosa* formed about 11–15 MY ago (mean dS = 0.39, 0.52; 11.3 MY, 15.0 MY). The inferred ages of neo-sex chromosomes are in between those for the neo-sex chromosomes of *D*. *miranda*, whose older neo-sex chromosome (chromosome XR, Muller element D) fused to the ancestral X about 13–15 MY ago, and its neo-X/neo-Y (Muller element C) formed about 1.5 MY ago [20]. Note that the estimated age of neo-sex chromosomes may exceed the inferred speciation time even if they formed after speciation because of faster sequence evolution on the neo-Y chromosome. Selection is less efficient on the non-recombining neo-Y chromosome [20], and slightly deleterious synonymous mutations may thus accumulate faster; a higher mutation rate in males relative to females (male-driven evolution) could further increase the rate of neutral substitutions along the neo-Y branch [30].

### Neo-X chromosomes have acquired dosage compensation

**Fig 3:**
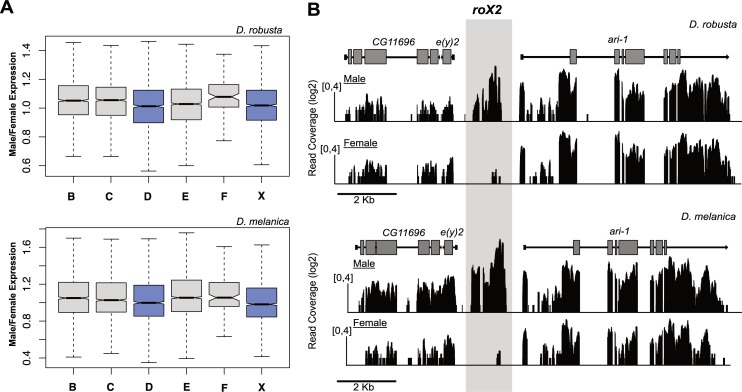
Dosage compensation of neo-X chromosomes. (A) We profiled gene expression in *Drosophila* heads using RNA-seq, and male/female gene expression ratios are shown for each chromosome for *D*. *robusta* (top) and *D*. *melanica* (bottom). X-linked genes (blue boxes) are expressed at roughly equal levels in both males and females, suggesting that both the ancestral and neo-X chromosomes are fully dosage compensated. Underlying data can be found in S3 Data. (B) Identification of *roX2*. In other *Drosophila* species, the male-specific noncoding *roX2* transcript is located between the protein-coding genes *e(y)2* and *ari-1*. We identified the *roX2* transcript in *D*. *melanica* and *D*. *robusta* by visualizing RNA-seq data from heads across the *e(y)2*–*ari-1* genomic region. The gray box indicates the location of *roX2*, which shows abundant expression only in males. RNA-seq, RNA sequencing.

Degeneration of the Y chromosome creates selective pressure to dosage compensate the X chromosome [31,32]. Our genomic analysis demonstrates that the neo-Y chromosomes of members of the *D*. *robusta* and *D*. *melanica* species group have very few genes left (**S2 Table**), and their neo-X chromosomes may thus have already acquired full dosage compensation. We gathered male and female RNA sequencing (RNA-seq) data from *D*. *melanica* and *D*. *robusta* heads to test whether expression levels of the newly formed neo-X chromosomes are similar between males and females (that is, whether they have evolved dosage compensation). Dosage compensation is absent in testis of *Drosophila* [33,29], and genes expressed in gonads show a nonrandom distribution on sex chromosomes [34–37]; we thus chose heads (a somatic tissue) to test for dosage compensation on the neo-X of *D*. *melanica* and *D*. *robusta*. We assigned *D*. *melanica* and *D*. *robusta* genomic scaffolds to chromosomes based on homology to *D*. *virilis* and identified genes directly from RNA-seq alignments to those same genomic scaffolds. Fig 3A shows male/female expression ratios for genes on the different chromosomes. Genes located on the neo-X in both species show very similar male/female expression ratios to genes on the ancestral X, suggesting that they have evolved full dosage compensation. Interestingly, however, genes located on the ancestral X as well as the neo-X in both species show slightly higher expression in females than males compared to autosomes (**Fig 3A**). This is consistent with the female-biased expression pattern of X chromosomes previously observed in *Drosophila* [34,35].

### Annotation of *roX* sequences and identification of MSL-binding sites

Comparative studies in *Drosophila* have shown that dosage compensation by the MSL complex is conserved across species [15,16,38,39]. Moreover, newly formed neo-X chromosomes evolve dosage compensation by acquiring novel MSL-binding sites that are able to recruit the MSL complex [38,39]. In *D*. *melanogaster*, two noncoding RNAs (*roX1* and *roX2*) are part of the MSL complex, and a recently developed technique known as ChIRP has been successfully used to map MSL binding in several *Drosophila* species by isolating and sequencing DNA bound by the *roX* noncoding RNAs [38] (see **Fig 1C**).

Noncoding RNAs evolve quickly at the DNA sequence level but can be identified based on microsynteny and their male-specific expression [38]. Previous work has shown that while *roX1* strongly localizes to the X chromosome in *D*. *melanogaster*, it shows much weaker X localization in other species of *Drosophila* (including *D*. *virilis*) [38]. *RoX2*, on the other hand, shows strong localization to the X chromosome in all *Drosophila* species studied so far [38] and has male-specific expression in dozens of species across the *Drosophila* phylogeny [38]; we thus focused on identifying *roX2* in our target species. In *D*. *virilis*, *roX2* is located between the protein-coding genes *ari-1* and *e(y)2*. To identify the *roX2* locus, we first searched each genome for synteny blocks likely containing *roX2* based on the conserved location of *ari-1* and *e(y)2* homologs and then mapped RNA-seq data from *D*. *melanica* and *D*. *robusta* males and females in order to identify genomic regions showing male-specific expression (**Fig 3B**). Indeed, we found male-specific RNA-seq reads from our candidate region in both species, and we assembled the RNA-seq reads mapping to this location to generate the full-length *roX2* transcripts from each species. We identified *roX2* in *D*. *nigromelanica* based on homology to the *D*. *melanica* and *D*. *robusta* transcripts (**S1 Fig**).

**Fig 4:**
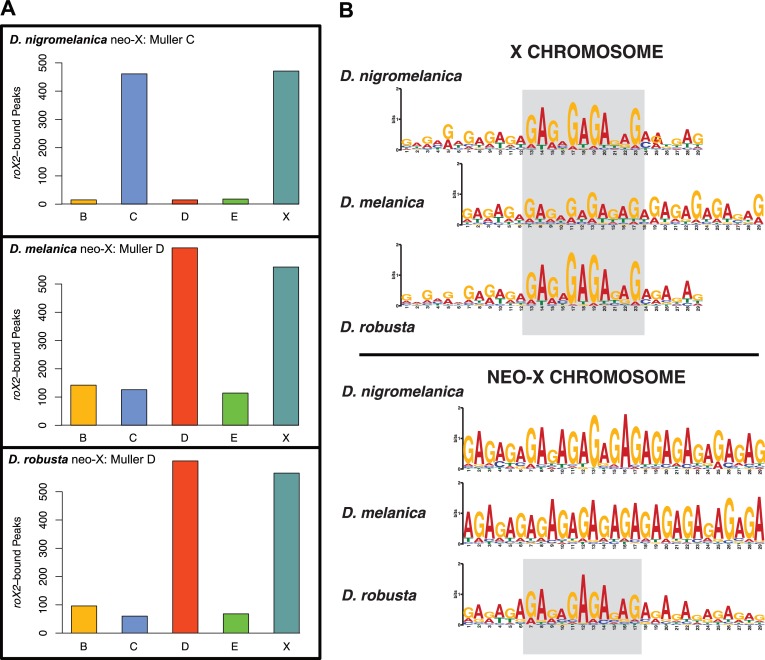
*roX2* binding locations and sequence motifs. (A) *RoX2*-bound regions are highly enriched on both the ancestral X chromosome and the neo-X chromosome in all three species. Underlying data can be found in S4 Data. (B) Identification of MSL binding motif. From the strongly bound peaks corresponding to CESs, we identified a GA-rich sequence motif on both the ancestral X and neo-X chromosome in all three species. The gray boxes highlight the conserved core motif, which is very similar to that found in *D*. *melanogaster*. CES, chromatin entry site; MSL, male-specific lethal.

To map MSL-binding sites, we designed nonoverlapping oligos against *roX2* in *D*. *nigromelanica*, *D*. *melanica*, and *D*. *robusta* using a split oligo design [38]. We performed two independent ChIRP experiments with different nonoverlapping oligo sets (**S3 Table**) and generated 100-bp paired-end (PE) sequencing libraries for each oligo set as well as an input control. We aligned the sequencing reads to their respective genomes and identified genomic regions showing *roX2* enrichment peaks. We found that *roX2* binding is highly correlated between independent probe sets for each of the species **(S2 Fig**), and we identified MSL-binding sites as overlapping peaks between the two independent ChIRP experiments. We identified between 980 and 1,570 peaks in each species, with the majority located on either the X or neo-X chromosome (approximately 75%–95%, **Fig 4A**). As done previously [38,39], we set an enrichment threshold (see Materials and methods) to identify the subset of the most strongly bound peaks as CESs. We found similar numbers of CESs on the ancestral X (212–295) and the neo-X (258–290), suggesting that the neo-X chromosomes in each species have evolved full dosage compensation (**Table 1**). Note that the slightly higher number of CESs on the neo-X of *D*. *melanica* and *D*. *robusta* compared to their ancestral X could be due to the larger assembled size of their neo-X (the neo-X assembly is roughly 10% larger in both species relative to the ancestral X). Indeed, the density of CESs is similar for the ancestral X and the neo-X in both species and similar to that of the ancestral X in *D*. *nigromelanica* (that is, a CES every 58–68 kb; **S4 Table**). This is consistent with gene expression patterns, and the number of CESs per chromosome are similar to what has been found in other species with fully dosage-compensated X chromosomes [13,38]. A slightly lower density of CESs on the neo-X of *D*. *nigromelanica* (a CES every 83 kb; **S4 Table**), on the other hand, may reflect the evolution of incomplete dosage compensation on this more recently formed neo-X, as has been found on the young neo-X chromosome of *D*. *miranda* [39].

**Table 1:** CESs identified in *D*. *nigromelanica*, *D*. *melanica*, and *D*. *robusta*.

*Drosophila* species	X chromosome			Neo-X chromosome		
	MRE	Pion-X site	No motif	MRE	Pion-X site	No motif
*D*. *nigromelanica*	174 (59%)	84 (27%)	37 (13%)	137 (53%)	73 (28%)	48 (19%)
*D*. *melanica*	175 (64%)	65 (24%)	35 (13%)	169 (58%)	82 (28%)	39 (13%)
*D*. *robusta*	139 (66%)	57 (27%)	16 (8%)	157 (59%)	86 (32%)	25 (9%)

**Abbreviations**: CES, chromatin entry site; MRE, MSL recognition element; MSL, male-specific lethal; pion-X site, pioneering site on the X.

We used the software package MEME [40] to identify motifs enriched within *roX2*-bound CES regions. Molecular studies in *D*. *melanogaster* identified a GA-rich sequence motif that is targeted by the MSL complex [39]. Consistent with previous studies, we find a GA-rich sequence motif to be highly enriched on the ancestral X in every species studied, and the same GA-rich sequence motif is found on the newly evolved X chromosomes in species of the *D*. *robusta* and *D*. *melanica* species group (**Fig 4B, S5 Table**). A subset of CESs, called pioneering sites on the X (pion-X sites), are thought to be responsible for the initial recruitment of the MSL complex to the X chromosome [41]. Pion-X sites share the low-complexity GA-rich motif with canonical CESs (that is, they are generally a subset of MRE motifs) but contain a more complex CAC 5´ extension [41]. We searched each CES to identify matches to both the canonical MRE motif and the pion-X site motif (**S3 Fig, S5 Table**). Between 85%–91% of the CES sites identified contain either an MRE and/or pion-X site motif on both the ancestral X and on the neo-X (174/175/139 MRE and 84/65/57 pion-X sites on the ancestral X and 137/169/157 MRE and 73/82/86 pion-X sites on the neo-X in *D*. *nigromelanica*/*D*. *melanica*/*D*. *robusta*; **Table 1**). Thus, this confirms that the molecular machinery for dosage compensation is conserved in *Drosophila* and that the MSL complex has been independently recruited to transcriptionally up-regulate newly formed X chromosomes by acquisition of the MSL binding motif.

### CES conservation and turnover on the ancestral X

Contrasting CES evolution on homologous chromosomes that either ancestrally or convergently evolved dosage compensation allows us to study evolutionary patterns and constraints of CES conservation, acquisition, and turnover [21,22,38,39]. In particular, the ancestral X was fully dosage compensated by the MSL complex in the last common ancestor of *D*. *melanica*, *D*. *nigromelanica*, and *D*. *robusta*, and contrasting CES locations on the ancestral X chromosome can inform us of the evolutionary stability and turnover of shared CESs. In contrast, chromosome 3L independently evolved dosage compensation in *D*. *melanica* and *D*. *robusta*, and convergent acquisition of CESs on homologous positions may reflect either the presence of dosage-sensitive genes in a particular location and a strong need to evolve CESs or the presence of presites (that is, nucleotide sequences that resemble the MRE motif) and thus an easy mutational path to acquire CESs.

Overall, we find 72 CESs (about 28%) and 46 motifs (20%) to be syntenic between all three species on the ancestral X chromosome, and 50% of CESs (and 41% of motifs) are shared between the more closely related *D*. *melanica* and *D*. *nigromelanica* species pair (**S6 Table**). Each species from the *D*. *melanica* subgroup shares about 43% of its CESs with the more distantly related *D*. *robusta*. Inspection of species-specific CESs reveals that the majority of orthologous regions in other species (77% on average) tend to be bound by *roX2*, but at too low a level to pass our genome-wide threshold for CES identification; the majority of orthologous regions also contain the MRE/pion-X site motif (73% on average), suggesting that most CESs on the ancestral X are conserved between species.

On the other hand, about 25% of CESs evolved independently at syntenic positions on chromosome 3L in *D*. *melanica* and *D*. *robusta* (that is, 54 out of 251/243), suggesting that they arose from a presite present in their common ancestor. Indeed, for 90% of these sites, the orthologous region in *D*. *nigromelanica*, in which this chromosome is an autosome, shows homology to the MRE/pion-X site motif, indicating that the MSL-binding site evolved from a preexisting prebinding site. Five genomic regions evolved CESs independently in *D*. *melanica* and *D*. *robusta* by either GA expansions or insertions.

### Mutational paths to acquire MSL-binding sites on the neo-X

**Fig 5:**
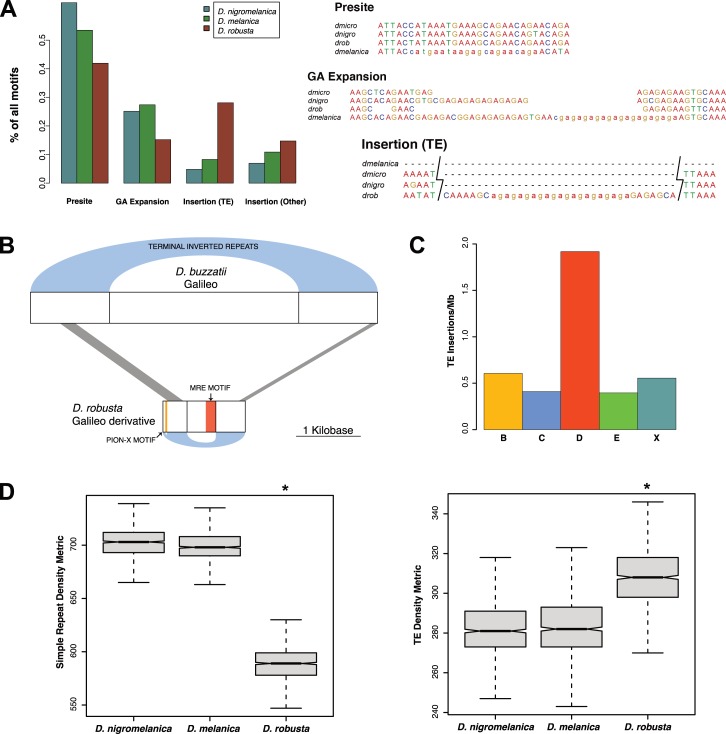
Evolution of CES motifs. (A) We used a comparative genomics approach to determine the evolutionary mechanism that gave rise to the MSL-binding motifs found within each CES on the neo-X chromosome of each species; shown is one example of each major type. *D*. *robusta* has a larger number of CES motifs derived from TE insertions compared to the other two species, whereas *D*. *nigromelanica* and *D*. *melanica* have a larger number of motifs that arose from GA expansion. Underlying data can be found in S5 Data. (B) We identified a Galileo-like TE that gave rise to at least 24 CES motifs on the *D*. *robusta* neo-X chromosome. (C) Insertions of this TE are significantly enriched on the *D*. *robusta* neo-X, compared to both autosomes and the ancestral X chromosome (binomial test *P* = 3.2 × 10^‐14^). Underlying data can be found in S5 Data. (D) Both *D*. *nigromelanica* and *D*. *melanica* genome assemblies have a higher density of simple repeats, whereas the *D*. *robusta* genome assembly has a higher density of TEs (Wilcoxon test *P* < 2.2 × 10^‐16^ for both comparisons). The repeat density metric refers to the number of 1-kb windows that overlap a repeat in the genome assembly out of 1,000 randomly placed windows. Underlying data can be found in S5 Data. CES, chromatin entry site; MRE, MSL recognition element; MSL, male-specific lethal; pion-X site, pioneering site on the X; TE, transposable element.

How are novel CESs acquired at the molecular level? Previous analysis of CES evolution in *Drosophila* revealed diverse mutational paths by which novel CESs originated in different fly species [21,22,38]. They include the use of prebinding sites (that is, sequence motifs that resemble the MRE motif and predate CES formation), simple GA expansions [42], or the spreading of CESs by TE mobilization [22]. Overall, we find that about 40%–60% of CESs on the newly formed neo-X chromosomes evolved from a presite. Thus, CESs often evolve from sequences that ancestrally resemble the MRE or pion-X site motif, and variability in sequence composition within similar GA-rich motifs [43] or changes to either the flanking sequences of CESs [44] or the repeat composition of the X [45], or possibly changes to the 3D organization [46], may allow these sequences not previously targeted by the MSL complex to function as CESs. In *D*. *melanica* and *D*. *nigromelanica*, most remaining CESs (approximately 25%) are created by simple GA expansions, whereas in *D*. *robusta*, 28% of novel CESs were created by TE insertions (**Fig 5A and 5B**). Thus, all three species utilized diverse mutational paths to evolve hundreds of novel CESs on their independently formed neo-X chromosome.

We also compared the mechanisms that gave rise to MRE motifs versus those that produced pion-X site motifs separately (**S4 Fig**). The relative frequencies of each mutational mechanism were similar for both types of motifs, with the exception that MRE motifs were much more likely to arise from GA expansions compared to pion-X site motifs (Fisher’s exact test *P* = 4.9 × 10^‐8^), consistent with the more complex sequence content of the pion-X site motif. A recent study suggested that CESs might originate by the co-option of GA-rich polypyrimidine tracts that are located at the 3´ 100 bp of introns and are used for splicing [38]. We found that 7%–12% of neo-X CES motifs are within the 3´ 100 bp of introns, the expected location for motifs arising from a co-opted polypyrimidine tract [38]. Both MRE and pion-X site motifs arose from these locations (55%–76% MRE, 24%–45% pion-X sites), and in roughly half of these cases, the polypyrimidine tract served as a ready-made motif (that is, presite), whereas in the remaining half, there were additional GA-expansion mutations, suggesting that the co-option of these features can involve multiple mutational paths (**S5 Fig**). The majority of MSL-binding motifs within CESs on neo-X chromosomes lie within gene bodies (introns and UTRs; **S6 Fig**), consistent with dosage compensation up-regulating gene expression [38].

We investigated the *D*. *robusta* TE-derived motifs in more detail and found that both MRE and pion-X site motifs were derived from a total of 18 different families (**S7 Table**). While most of these TE families were associated with only a single motif, we also identified a single family of elements that gave rise to 24 MSL binding motifs (17 MRE motifs and 7 pion-X site motifs). This TE family contains terminal inverted repeats with weak homology to those of two related galileo elements that have been identified in *D*. *buzzatii* and *D*. *melanogaster* (**Fig 5B**). The TE consensus sequence contains a match to the pion-X site motif near its 5´ end as well as an MRE motif near its 3´ end. We identified several hundred fragmented copies of this TE in our *D*. *robusta* genome assembly. These copies are highly enriched on the *D*. *robusta* neo-X chromosome (binomial test *P* = 3.2 × 10^‐14^, **Fig 5C**) and overlap CESs more often than expected by chance (Fisher’s exact test *P* < 2.2 × 10^‐16^). Of the 66 copies that are located on scaffolds that were assigned to Muller elements, 32 are from the neo-X chromosome, and 24 of these 32 copies lie within CESs. The average pairwise genetic distance between these copies is 0.47, which suggests they were active around the time when the neo-sex chromosomes of *D*. *robusta* were formed (mean dS: 0.39). Thus, enrichment of this TE on the neo-X of *D*. *robusta*, its occurrence within CESs and the presence of a binding motif for the MSL complex, and its inferred time of mobilization around the formation of neo-sex chromosomes strongly suggests that this TE was actively involved in dispersing MSL-binding sites along the neo-X.

### Genomic contingencies and heterogeneity in binding site evolution

What drives heterogeneity in CES acquisition across lineages? While *D*. *melanica* and *D*. *nigromelanica* evolved most novel MSL binding motifs by GA expansions, *D*. *robusta* utilized a TE for acquiring novel CESs. To investigate whether certain genomic factors prime a genome to preferentially evolve CESs by a particular mutational path, we analyzed the content of repetitive DNA—both microsatellite and TE density—in the different lineages. Interestingly, we found that *D*. *melanica* and *D*. *nigromelanica* differ in their overall repeat composition from *D*. *robusta*: they both show a higher density of simple repeats but a lower density of TE sequences (Wilcoxon test *P* < 2.2 × 10^‐16^ for both comparisons) (**Fig 5D**). Higher TE density in *D*. *robusta* is consistent with its larger assembled genome size (185 Mb in *D*. *robusta* versus 150 Mb in *D*. *melanica* and 164 Mb in *D*. *nigromelanica*). Thus, this observation is consistent with the notion that historical contingencies constrain evolutionary patterns of MSL-binding–site evolution. TEs may be more often utilized for rewiring regulatory networks in species with a higher number of TEs, but a larger TE burden may also contribute to increased genome sizes. On the other hand, a higher density of simple satellites in *D*. *melanica* and *D*. *nigromelanica* may have preadapted them to evolve novel MRE sites by GA-sequence expansion.

## Discussion

We took advantage of naturally occurring variation in sex chromosome karyotype in *Drosophila* species to study independent replicates of solving the same evolutionary challenge: to dosage compensate newly formed neo-X chromosomes by acquiring hundreds of MSL-binding sites in response to Y degeneration.

The independent acquisition of dosage compensation in *Drosophila* allows us to address several important questions in evolutionary biology and gene regulation: first, how repeatable is evolution? Evolutionary biologists have long debated the predictability of the evolutionary process. At one extreme, evolution could be highly idiosyncratic and unpredictable, since the survival of the fittest could occur along a great number of forking paths. Alternatively, constraints on evolution may force independent lineages to frequently converge on the same genetic solutions for the same evolutionary challenge. Second, how do regulatory networks evolve? And what is the contribution of TEs to regulatory evolution? Evolutionary innovations and adaptations often require rapid and concerted changes in regulation of gene expression at many loci [47]. TEs constitute the most dynamic part of eukaryotic genomes, and the dispersal of TEs that contain a regulatory element may allow for the same regulatory motif to be recruited at many genomic locations, thereby drawing multiple genes into the same regulatory network [48–50]. Third, what makes a binding motif functional? The genomes of complex organisms encompass megabases of DNA, and regulatory molecules must distinguish specific targets within this vast landscape. Regulatory factors typically identify their targets through sequence-specific interactions with the underlying DNA, but they typically bind only a fraction of the candidate genomic regions containing their specific target sequence motif. An unresolved mystery in regulatory evolution is what drives the specificity of binding to a subset of genomic regions that all appear to have a sequence that matches the consensus binding motif.

Several features make dosage compensation in *Drosophila* a promising system to tackle these questions. The genetic architecture for most adaptations—especially those involving regulatory changes—as well as the timing and exact selective forces driving them is generally little understood. In contrast, we have detailed knowledge of the molecular mechanism of dosage compensation in *Drosophila*. We know the *cis*- and *trans*-acting components of this regulatory network and the regulatory motif for targeting the MSL complex to the X. We have clear expectations of which genomic regions should acquire dosage compensation and about the timing and the evolutionary forces that drive wiring of hundreds of genes into the dosage-compensation network on newly evolved X chromosomes. Specifically, Y degeneration is a general facet of sex chromosome evolution, creating selective pressures to up-regulate X-linked genes in males. Dosage compensation should thus only evolve on neo-X chromosomes whose neo-Y homologs have started to degenerate and should evolve simultaneously or shortly after substantial gene loss has occurred on the neo-Y [31,32]. Indeed, comparative data in *Drosophila* support this model of dosage-compensation evolution. *Drosophila* species with partially eroded neo-Y chromosomes exist that have not yet evolved MSL-mediated dosage compensation, including *D*. *busckii* [25] and *D*. *albomicans* [51,52], lending empirical support to the notion that dosage compensation evolves in response to Y degeneration and not the other way round. Thus, our refined understanding of how, when, why, and where dosage compensation in *Drosophila* evolves makes this an ideal model system to study the repeatability of evolution and the evolution of regulatory networks.

### How predictable is evolution?

Results from evolution experiments indicate that although evolution is not identical in replicate populations, there is an important degree of predictability [53]. Experimentally evolved populations under controlled, identical conditions consistently show parallelism in which mutations in certain genes are repeatedly selected [3,4]. However, organisms adapting to similar environments are not genetically identical, but their genome instead carries the legacy of their unique evolutionary trajectory, raising the question of how genomic differences affect genetic parallelism.

Sex chromosome–autosome fusions have independently created neo-sex chromosomes in different *Drosophila* lineages. This provides us with several independent replicates to study how, on the molecular level, evolution has solved the same adaptive challenge: acquiring hundreds of binding sites to recruit the MSL complex to newly formed X chromosomes. This allows us to quantify how much variation there is, both within and between species, in the underlying mutational paths to acquire hundreds of MSL-binding sites on neo-X chromosomes and identify genomic contingencies that will influence the repeatability of evolutionary trajectories. Importantly, neo-sex chromosomes of *Drosophila* are evolutionarily young (between 0.1–15 MY old), which allows us, in many cases, to infer the causative mutations that have resulted in the gain of a regulatory element and decipher the evolutionary processes at work to draw hundreds of genes into a new regulatory network.

Our results suggest that the evolution of MSL-binding sites is highly opportunistic but contingent on genomic background. In particular, we find that each independently evolved neo-X chromosome uses a diverse set of mutational pathways to acquire MSL-binding sites on a new neo-X chromosome, ranging from microsatellite expansions to the utilization of presites to TE insertions. However, different lineages differ with regards to the frequency of which mutational paths are most often followed to acquire novel binding sites, and this propensity may depend on the genomic background. In particular, we find that the two species with the higher density of simple repeats are more prone to utilize expansions in GA microsatellites to gain a novel MSL-binding site. In contrast, *D*. *robusta* has an elevated TE density compared to its sibling species, and we find that the dispersal of a TE has played an important role in the acquisition of MSL-binding sites on its neo-X chromosome. Thus, this suggests that the genomic background of a species predisposes it to evolve along a particular path, yet the evolutionary process is random and resourceful with regards to utilizing a variety of mutations to create novel MSL-binding sites. However, as discussed in the introduction, different phenotypes show drastic differences in their underlying genetic architecture, and the importance of genomic background likely differs among traits and species [2].

### The importance of TE-mediated regulatory rewiring

Evolutionary innovations and adaptations often require rapid and concerted changes in regulation of gene expression at many loci [47]. It has been suggested that TEs play a key role in rewiring regulatory networks, since the dispersal of TEs that contain a regulatory element may allow for the same regulatory motif to be recruited at many genomic locations [48–50]. A handful of recent studies have implicated TEs as drivers of key evolutionary innovations, including placentation in mammals [54] or rewiring the core regulatory network of human embryonic stem cells [55]. While these studies demonstrate that TEs can, in principle, contribute to the creation or rewiring of regulatory networks, they do not address the question of how often regulatory elements evolve by TE insertions versus by other mutations. That is, the importance of TEs in contributing to regulatory evolution is not known. Quantification of the role of TEs would require a priori knowledge of how and when regulatory networks evolve and a detailed molecular understanding of which genes are being drawn into a regulatory network and how. As discussed above, these parameters are well understood for dosage compensation in flies.

Our previous work in *D*. *miranda* has shown that a helitron TE was recruited into the dosage-compensation network at two independent time points. The younger 1.5-MY-old neo-X chromosome of *D*. *miranda* is in the process of evolving dosage compensation, and dozens of new CESs on this chromosome were created by insertions of the ISX element [22]. We showed that the domesticated ISX TE gained a novel MRE motif by a 10-bp deletion in the ISY element, which is a highly abundant TE in the *D*. *miranda* genome [22]. We also found the remnants of a related (but different) TE at CES on the older neo-X of this species (which formed roughly 13–15 MY ago), but the TE was too eroded to reconstruct its evolutionary history. Here, we identified another domesticated TE that was utilized to deliver MSL-binding sites to a newly formed neo-X chromosome, but no significant TE contribution was found for MSL-binding site evolution in two independent neo-X chromosomes.

Our data shed light on the question of when we expect TEs to be important in regulatory evolution. For TEs to contribute to regulatory rewiring, two conditions have to be met: a regulatory element (or a progenitor sequence that can easily mutate into the required binding motif) needs to be present in the TE, and that TE needs to be active in the genome (and not yet silenced by the host machinery). TEs undergo a characteristic life cycle in which they invade a new species (or escape the genome defense by mutation) and transpose until they are silenced by the host genome [56]. Once a TE is robustly repressed, it no longer can serve as a vehicle to disperse regulatory elements, so the time window when a particular TE family can be domesticated is probably short and needs to coincide with a necessity to disperse regulatory motifs. A high TE burden does increase that chance, but at a cost: maintaining active TEs in the genome allows a rapid response to evolutionary challenges but also creates a major source of genomic mutation, illegitimate recombination, genomic rearrangements, and genome size inflation [57].

Our findings support this view of a TE tradeoff. The ISY element in *D*. *miranda* is the most highly abundant transposon in the *D*. *miranda* genome and is massively contributing to the degeneration of the neo-Y in this species [26]. Indeed, our genomic analysis has revealed >20,000 novel insertions of the ISY element on the neo-Y, often within genes [26]. Yet, it contained a sequence that was only one mutational step away from a functional MSL-binding site (that is, a single 10-bp deletion), and domestication of this element allowed for the rapid dispersal of functional binding sites for the MSL complex along the neo-X. The domestication of the TE in *D*. *robusta* occurred too long ago for us to reconstruct its exact evolutionary history and the potential damage its mobilization may have caused while it was active. However, consistent with a tradeoff that the host genome faces, we find that *D*. *robusta* has a higher TE density than its sister species and also a considerably larger genome size, yet a TE contributed to wiring hundreds of genes into the dosage-compensation network on its neo-X.

### What makes a binding site functional?

Perhaps surprisingly, in many instances, we are unable to detect specific mutations that would generate a novel MSL binding motif. Instead, we find that functional MSL-binding sites are derived from presites containing the GA-rich motif that was already present in an ancestor in which the neo-X is autosomal and in which these sequences do not recruit the MSL complex. The MSL binding motif is only modestly enriched on the X chromosome compared to the autosomes (only approximately 2-fold), and only a small fraction of putative binding sites are actually bound by the MSL complex [13]. The dosage-compensation machinery shares this characteristic with many other sequence-specific binding factors whose predicted target motifs are often in vast excess to the sites actually utilized. It has been speculated that other genomic aspects, such as chromatin context or the 3D organization of the genome, could help to distinguish between utilized and nonutilized copies of a motif. Our finding that a large number of sites can acquire the ability to recruit the MSL complex, without any apparent associated changes at the DNA level, supports the view that epigenetic modifications or changes to the 3D architecture of the genome help to ultimately determine which putative binding sites in the genome are actually utilized [44,46]. In *D*. *melanogaster*, the X chromosome has a unique satellite DNA composition, and it was suggested that these repeats play a primary role in determining X identity during dosage compensation [45]. Furthermore, localization of the MSL complex to MREs is dependent on an additional cofactor, the CLAMP protein [58]. CLAMP binds directly to GA-rich MRE sequences and targets MSL to the X chromosome but also binds to GA-rich sequence elements throughout the genome [58]. Recent work has shown that variability in sequence composition within similar GA-rich motifs drive specificity for CLAMP binding [43], and variation within seemingly similar *cis* elements may also drive context-specific targeting of the MSL complex. Future investigations of changes in the chromatin level, the repeat content, and the genomic architecture of these newly formed sex chromosomes will help to resolve this outstanding question.

## Materials and methods

### Genome sequencing, assembly, and alignment

DNA was extracted from single flies using the Qiagen PureGene Kit (Qiagen, Hilden, Germany), and two PE Illumina sequencing libraries (male and female) were prepared for each species. The Illumina Nextera library prep kit (Illumina, San Diego, CA, USA) was used for *D*. *melanica* and *D*. *robusta* (150-bp PE reads), while the Illumina TruSeq kit (100-bp PE reads; Illumina) was used for the remaining species. Genome assemblies were generated for males and females separately by first error-correcting reads using *BFC* [59] and then assembling the corrected reads using *IDBA* [60]. A whole-genome alignment was constructed using the female assemblies for the five species studied here plus *D*. *virilis* using *Mercator* [61].

### Phylogeny

To create a whole-genome phylogeny, the *D*. *virilis* genome was split into 250-bp windows. Each window was extracted from the *Mercator* whole-genome alignment, and windows were retained if the aligned sequence from each species contained no more than 10% of positions as gaps. Retained windows were further filtered to ensure that each window was at least 1 kb from the closest neighboring window. These windows were concatenated to produce a multiple-sequence alignment containing 1.1 million positions. The RAxML rapid bootstrapping algorithm [62] was used to produce a maximum likelihood phylogeny from this alignment.

### Chromosome assignments and sex linkage

Chromosome assignments for *D*. *virilis* scaffolds were obtained from [63]. The scaffolds from each species studied here were assigned to Muller elements based on their alignment to *D*. *virilis* scaffolds from the *Mercator* whole-genome alignment.

To determine which Muller elements are X linked in each species, male and female Illumina reads were aligned separately to the female genome assemblies using *bowtie2* [64], and male/female coverage ratios were calculated for each female scaffold.

Y-linked scaffolds were identified from the male assemblies using *YGS* [65].

### RNA-seq and *roX2* identification

For each sex, heads were removed from five flies, flash frozen in liquid nitrogen, and placed into Trizol for RNA extraction. The Illumina TruSeq RNA kit was used to prepare unstranded, single-end 50-bp sequencing libraries for each sex. RNA-seq data were aligned to the female reference genome assembly using *Hisat2* [66], and gene models were generated from the merged male + female spliced alignments, along with normalized expression values, using *StringTie* [67]. Male and female RNA-seq read coverage was used to identify the location of *roX2* in *D*. *melanica* and *D*. *robusta* (see **Fig 3B**), and *roX2* transcripts were extracted from the genome assemblies based on the *StringTie* gene models. The *D*. *nigromelanica roX2* transcript was identified based on homology to the *D*. *melanica* transcript using *Exonerate* [68].

### Species pairs and neo-X/Y chromosome divergence

For each species, *D*. *melanogaster* peptides were searched against the set of neo-X- and Y-linked scaffolds using a translated BLAST search [69]. The resulting neo-X- and Y-linked gene models were further refined using *Exonerate*, and their coding sequence was aligned using the codon model in *PRANK* [70]. Ks values were calculated for each neo-X/Y pair using *KaKs_Calculator* [71]. For species divergences, orthologous genes were identified using the *D*. *robusta* gene models from *StringTie* and the *Mercator* whole-genome alignment. Refinement of gene models, alignment, and Ks values were obtained as described above.

### Divergence time estimates

We used a neutral mutation rate estimate of 3.46 × 10^‐9^ per base per generation, which was experimentally determined from *D*. *melanogaster* [28]. The species studied here have a generation time that is roughly twice as long as *D*. *melanogaster*, and we therefore used the lower bound of the estimate of the number of generations per year for Drosophilids (5 generations) [72] to convert the mutation rate to time-based units (1.73 × 10^‐8^ mutations per base per year).

### ChIRP

ChIRP sequencing libraries were prepared according to the published protocol [73] using the *Drosophila*-specific modifications described in [38]. For each species, ChIRP libraries were prepared from 2 different pools of 6 probes (**S3 Table**), which were tiled across the roX2 transcript. Input control libraries were also prepared for each species by extracting DNA from an aliquot of the cell lysate immediately prior to probe hybridization. Wandering third-instar larvae were used for *D*. *melanica* and *D*. *robusta*. Because of the difficulty in collecting sufficient larvae for *D*. *nigromelanica*, adult males were used instead. 100-bp PE Illumina reads were generated for each pool for each species and aligned to the female reference genome assembly using *bowtie2*. Peaks of roX2 binding were identified by running *MACS* [74] on each pool separately, along with the control library, and a final set of peaks was generated by retaining only the subset of peaks that were identified in both pools.

### Identification of CESs

The ChIRP libraries varied in overall signal versus background, likely because of differences in the hybridization efficiency of the different probe sets. For each species, we calculated the average fold enrichment (treatment versus control) across all peaks as a measure of overall ChIRP signal. The *D*. *robusta* libraries showed the highest signal, and we used the same enrichment threshold (20) that was previously used to identify CESs from *roX2* peaks [38]. For the remaining species, we identified CESs by scaling the enrichment threshold in proportion to our measure of ChIRP signal.

### Motif identification and evolution

We defined the location of CESs as a 500-bp region centered on the summit of the *roX2*-bound peak. We extracted the sequence from these regions for the ancestral and neo-X chromosomes separately and used the ZOOPS model in *MEME* [40] to identify enriched sequence motifs. We used *FIMO* [40] to determine the location of MRE and pion-X site motifs within each CES and assigned each CES as containing either an MRE motif or a pion-X site motif (whichever match had a higher score) or no motif (if there was no match to either motif). We used the *Mercator* whole-genome alignment to assess orthology of CES as well as individual motifs. For each neo-X CES, we manually viewed the alignment of its sequence motif with the orthologous sequences from the other five species to determine the mutational mechanism that gave rise to the motif.

### TE identification

De novo TE identification was performed for each species using *RepeatModeler* (https://github.com/rmhubley/RepeatModeler). To identify the genomic locations of TE families, *RepeatMasker* (https://github.com/rmhubley/RepeatMasker) was used with the *RepeatModeler* consensus sequences as the repeat library. *D*. *robusta* TEs that overlapped CESs were further classified using *CENSOR* [75] and *RepBase* [76].

### Repeat density estimates

For each species, we used *RepeatMasker* to separately identify simple repeats and TEs. Because the percentage of the genome assembly that falls into these two categories will be affected by differences in total assembly size between species, we used an alternative approach for determining the density of these repeat classes. For each species, we permuted the location of 1,000 1-kb windows for 1,000 permutations. For each iteration, we determined the number of windows that overlapped a simple repeat and the number of windows that overlapped a TE, which we termed the “repeat density metric.”

## Supporting information

S1 Fig We identified the *roX2* transcript in *D. melanica* and *D. robusta* by using gene synteny and RNA-seq data. The *D*. *nigromelanica roX2* transcript was identified based on sequence homology to the transcripts from *D*. *melanica* and *robusta*. A multiple sequence alignment of the three transcripts is shown here. RNA-seq, RNA sequencing.
(EPS)

S2 Fig For each species, two ChIRP-seq libraries were prepared using two pools of nonoverlapping probes tiled across the *roX2* transcript. Each dot in the scatterplot represents a *roX2*-bound peak identified in one or both pools, and its location in the plot reflects the fold enrichment (ChIRP/input control) of that region in each pool. ChIRP, Chromatin Isolation by RNA Purification; ChIRP-seq, ChIRP sequencing.
(TIFF)

S3 Fig We assigned each CES as containing an MRE motif, a pion-X site motif, or no motif, based on searching the consensus sequences of these motifs against the CES sequences and identifying the best-scoring match. To validate these matches, we extracted the MRE or pion-X site element from each assigned CES sequence and created a consensus logo for each X chromosome of each species. The consensus motifs are very similar to the MRE and pion-X site consensus motifs discovered in *D*. *melanogaster*. CES, chromatin entry site; MRE, MSL recognition element; MSL, male-specific lethal; pion-X site, pioneering site on the X.
(EPS)

S4 Fig Evolutionary mechanism that gave rise to the MRE or pion-X site motifs found within each CES on the neo-X chromosome of each species. Underlying data can be found in S6 Data. CES, chromatin entry site; MRE, MSL recognition element; MSL, male-specific lethal; pion-X site, pioneering site on the X.
(EPS)

S5 Fig For each species, we identified both MRE and pion-X site motifs in the 3´ 100 bp of introns, in which the GA-rich polypyrimidine tracts involved in RNA splicing are located. Shown here are combined counts for MRE and pion-X site motifs. Less than 15% of all motifs were derived from these tracts in any species. For those that did arise from polypyrimidine tracts, about half served as a ready-made motif (that is, presite), whereas in the other half, additional GA-expansion mutations occurred at the tract location. Underlying data can be found in S6 Data. MRE, MSL recognition element; MSL, male-specific lethal; pion-X site, pioneering site on the X.
(EPS)

S6 Fig For each neo-X chromosome CES, we identified the location of its MRE (or pion-X site) motif, in terms of the following genomic features: intron, CDS, UTRs, 500 bp upstream from gene start, and 500 bp downstream. We classified any motif not overlapping those features as intergenic. When considering all neo-X motifs (Panel A), for each species, we find that the majority lie within gene bodies in either introns or UTRs. When considering only the motifs that evolved from presites (Panel B), we see a similar pattern. Underlying data can be found in S6 Data. CDS, coding sequence; CES, chromatin entry site; MRE, MSL recognition element; MSL, male-specific lethal; pion-X site, pioneering site on the X.
(EPS)

S1 Table Genomic data generated. (XLSX)

S2 Table Inferred protein divergence of homologous gene pairs identified on the neo-sex chromosomes of *D. nigromelanica*, *D. melanica*, *D. lacertosa*, and *D. robusta*. (XLSX)

S3 Table Oligos used for roX2 ChIRP in *D. nigromelanica*, *D. melanica*, and *D. robusta*. (XLSX)

S4 Table Length and density of CESs on the different X and neo-X chromosomes of *D. nigromelanica*, *D. melanica*, and *D. robusta*. CES, chromatin entry site. (XLSX)

S5 Table CES location and motif identified (either MRE or pion-X site motif) in *D. nigromelanica*, *D. melanica*, and *D. robusta*. CES, chromatin entry site; MRE, MSL recognition element; MSL, male-specific lethal; pion-X site, pioneering site on the X. (XLSX)

S6 Table Synteny of CESs on the ancestral X chromosome across species. CES, chromatin entry site.
(XLSX)

S7 Table TEs contributing to CES evolution in *D. robusta*. CES, chromatin entry site; TE, transposable element. (XLSX)

S1 Data Ka and Ks values for all neo-X/Y gene pairs in *D. nigromelanica*, *D. melanica*, *D. robusta*, and *D. lacertosa*. Ks values are plotted in Fig 2B. (XLSX)

S2 Data Ratio of male/female Illumina sequencing coverage for genomic libraries aligned to the female assemblies for *D. nigromelanica*, *D. melanica*, *D. micromelanica*, *D. robusta*, and *D. lacertosa*, and assignment of scaffolds to Muller elements. Ratios are plotted in Fig 2C. (XLSX)

S3 Data Genome-wide expression values (using RNA-seq) for male and female *Drosophila* heads and chromosomal location of genes for *D. robusta* and *D. melanica*. Male/female gene expression ratios are plotted in Fig 3A. RNA-seq, RNA sequencing.
(XLSX)

S4 Data *Rox2*-bound regions on different chromosomes for *D. nigromelanica, D. melanica*, and *D. robusta*. Data are plotted in Fig 4A.
(XLSX)

S5 Data Relative contribution of different evolutionary mechanism that gave rise to the MSL-binding motifs found within each CES on the neo-X chromosome of *D. nigromelanica, D. melanica*, and *D. robusta*. Data are plotted in Fig 5A. Density of the galileo-like TE that gave rise to multiple CES motifs on the *D*. *robusta* neo-X across the genome. Data are plotted in Fig 5C. Density of simple repeats and TEs in *D*. *nigromelanica*, *D*. *melanica*, and *D*. *robusta*. Data are plotted in Fig 5D. CES, chromatin entry site; MSL, male-specific lethal; TE, transposable element.
(XLSX)

S6 Data Evolutionary mechanism that gave rise to the MRE or pion-X site motifs found within each CES on the neo-X chromosome of *D. nigromelanica, D. melanica*, and *D. robusta*. Data are plotted in S4 Fig. Percentage of (combined) MRE and pion-X site motifs located in the 3´ 100 bp of introns and the evolutionary mechanism that gave rise to them in *D*. *nigromelanica*, *D*. *melanica*, and *D*. *robusta*. Data are plotted in S5 Fig. Location of the MRE (or pion-X site) motif for each neo-X chromosome CES, in terms of the following genomic features in *D*. *nigromelanica*, *D*. *melanica*, and *D*. *robusta*: intron, CDS, UTRs, 500 bp upstream from gene start, and 500 bp downstream. We classified any motif not overlapping those features as intergenic. Data are plotted in S6 Fig. CDS, coding sequence; CES, chromatin entry site; MRE, MSL recognition element; MSL, male-specific lethal; pion-X site, pioneering site on the X.
(XLSX)

## Abbreviations

CES: chromatin entry site
ChIRP: Chromatin Isolation by RNA Purification
dS: synonymous site divergence
MRE: MSL recognition element
MSL: male-specific lethal
MY: million years
PE: paired end
pion-X site: pioneering site on the X
RNA-seq: RNA sequencing
TE: transposable element
