# Supplementary figures and images for: Contingency in the convergent evolution of a regulatory network: Dosage compensation in Drosophila
Source: PLoS Biol. 2019 Feb 11;17(2):e3000094. doi: 10.1371/journal.pbio.3000094 (PMC6417741; doi:10.1371/journal.pbio.3000094)

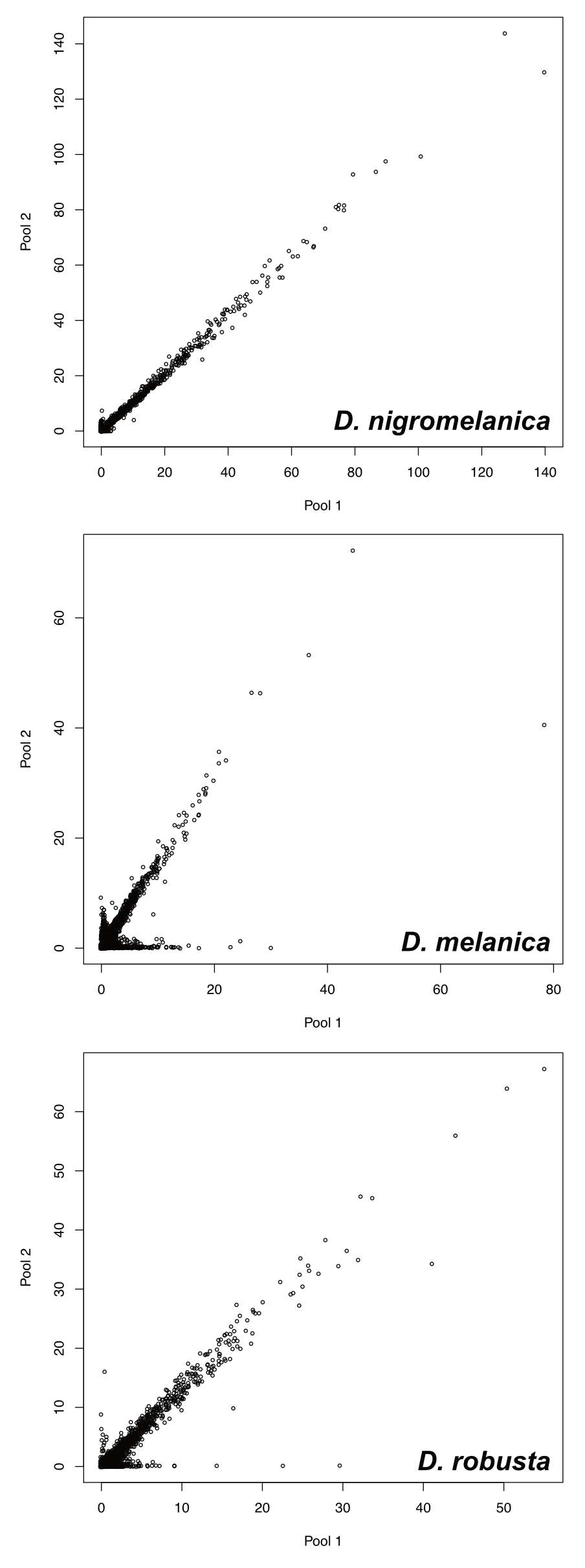

Supplement: S2 Fig — Each dot in the scatterplot represents a roX2-bound peak identified in one or both pools, and its location in the plot reflects the fold enrichment (ChIRP/input control) of that region in each pool. ChIRP, Chromatin Isolation by RNA Purification; ChIRP-seq, ChIRP sequencing. (TIFF) [file pbio.3000094.s002.tiff]
